# Glycated CD59 is a potential biomarker for gestational diabetes mellitus

**DOI:** 10.3389/fendo.2024.1374253

**Published:** 2024-09-16

**Authors:** Wanying Wang, Chong Xu, Xiaofan Lu, Wei Cao, Tengzi Zuo, Ying Zhang, Huiling Zou, Yu Sun

**Affiliations:** Department of Endocrinology, The Affiliated Suqian Hospital of Xuzhou Medical University, Suqian, Jiangsu, China

**Keywords:** gestational diabetes mellitus, glycosylated CD59, biomarker, oral glucose tolerance test, diagnostic value

## Abstract

**Objective:**

To explore the diagnostic value of glycated CD59 (gCD59) in gestational diabetes mellitus (GDM).

**Methods:**

A total of 707 pregnant women who underwent the first visit in the obstetric outpatient clinic of the Affliated Suqian Hospital of Xuzhou Medical University from January 2022 to July 2023 were included, and were grouped according to the International Association of the Diabetes and Pregnancy Study Groups(IADPSG) diagnostic criteria, and finally 113 cases in the GDM group and 559 cases in the normal glucose tolerance (NGT) group were included, and the concentration of gCD59 was determined by enzyme-linked immunosorbent assay (ELISA). The baseline data characteristics of the two groups were compared, the risk factors for GDM were explored by multivariate binary logistic analysis, and the diagnostic value of gCD59 in predicting GDM was explored by receiver operating characteristic (ROC) curve analysis.

**Results:**

The level of gCD59 in the GDM group was significantly higher than that in the NGT group (1.49 SPU vs 0.87 SPU). Multivariate regression analysis showed that gCD59, diastolic blood pressure (DBP) and thyroid stimulating hormone (TSH) were independent risk factors for GDM.The area under the curve (AUC) of gCD59 for the diagnosis of GDM was 0.681 (95% *CI*: 0.583-0.717), with a sensitivity of 71.7% and a specificity of 58.3%. In combination with fasting glucose, gCD59 effectively diagnosed GDM with higher AUC of 0.871 (95% *CI*: 0.708-1.000).

**Conclusion:**

gCD59 is an independent risk factor for GDM and a good biomarker for the diagnosis of GDM.

## 1 Introduction

Gestational Diabetes Mellitus (GDM), representing one of the most prevalent complications encountered during pregnancy, is characterized by glucose metabolism abnormalities that emerge or are first recognized during pregnancy in women who have no prior history of diabetes or glucose intolerance (1). The concerning rise in the prevalence of GDM in recent years,GDM endangers maternal and fetal health by increasing the risk of type 2 diabetes in mothers after delivery and predisposing offspring to various metabolic disorders in later life (2). Accordingly, the effective management of GDM, especially through stringent blood glucose control during pregnancy, is vital to reduce adverse outcomes for both mothers and children (3, 4).

Current clinical standards, primarily the oral glucose tolerance test (OGTT), are routinely recommended for GDM diagnosis during the mid-second trimester (5, 6). Despite its extensive application, OGTT requires fasting and has a low repeatability (7, 8). Lack of a uniform protocol further limits its utility in the clinical setting. Diagnostic methods for GDM include the one-step screening method recommended by the International Association of the Diabetes and Pregnancy Study Groups (IADPSG), with fasting ≥ 5.1 mmol/l, 1 h ≥ 10.0 mmol/l, 2 h ≥ 8.5 mmol/l, and a diagnosis of GDM if one of these criteria is met. The American College of Obstetricians and Gynecologists (ACOG) recommends a two-step screening test, with fasting ≥5.3 mmol/L,1 h ≥ 10.1 mmol/L, 2 h ≥8.7 mmol/L, 3 h ≥7.8 mmol/L, two or more values met or exceeded a required to make the diagnosis (9). Therefore, straightforward and reliable diagnostic alternatives are in urgent need (10). In recent years, novel biological markers have shown great potential in precision diagnosis and treatment of GDM, including various molecular types such as adipokines (lipocalin, leptin, endolipin, resistin); inflammatory factors (CRP, interleukin 6, TNF-a), epigenetic markers, small molecule proteins, small molecule metabolites and so on. Many scholars have studied this, but their sensitivity and specificity are limited, and there are no guidelines recommending specific biological markers (**Table 1**).

**Table 1:** Biological markers associated with gestational diabetes.

Biomarkers	Type of study	GDM Diagnostic guidelines	Sample collection time	Number of participants	AUC	Sensibility	Specificity
HbA1c (11)	Retrospective cross-sectional study	WHO1999 or ADA/WHO 2013	27 ± 5wk	262	0.714	68.1%	63.2%
SHBG (12)	Prospective observational studies	American Diabetes Association	15wk	269	0.692	85.2%	37.1%
Hs-CRP (12)	Prospective observational studies	American Diabetes Association	15wk	269	0.739	89%	55.3%
miR-195-5p (13)	Retrospective case-control study	IADPSG	24-28w	204	0.845	73.69%	96.85%
serum iron and zinc (14)	Prospective cohort study	Carpenter and Coustan	14-20w	1033	/	80.6%	50.7%
Small HDL particles (15)	prospective cohort study	IADPSG	12.8-15.6w	439	0.710	/	/
Acylcarnitines (16)	prospective nested case–control study	IADPSG	<18w	75	0.934	/	/

CD59 is glycosylphosphatidylinositol (GPI)-anchored protein, with a molecular weight of 18-20 kDa, ubiquitously expressed in mammalian cells (17, 18). gCD59, a stable soluble form of the broadly expressed complement regulatory protein CD59, emerges under hyperglycemic conditions. It detaches from the cell membrane through the action of phospholipase and persists in blood and urine. Notably, its expression level exhibits a significant correlation with blood glucose levels (19, 20), positioning gCD59 as a potential biomarker for diagnosing GDM. Bogdanet found that mid-trimester gCD59 diagnosed GDM with an area under curve (AUC) of 0.65 and the value was more pronounced in subjects with a high body mass index (BMI) (21). In the first trimester, the Glosh study found significantly higher levels of gCD59 in the GDM group than in the control group using the two-step approach as a diagnostic criterion (22). Despite the promising utility of gCD59 indicated in various studies, its application in the Chinese population has not been extensively explored. This study aimed to elucidate the diagnostic value of gCD59 for GDM and associated risk factors in a Chinese cohort.

## 2 Materials and methods

### 2.1 Study subjects

**Figure 1:**
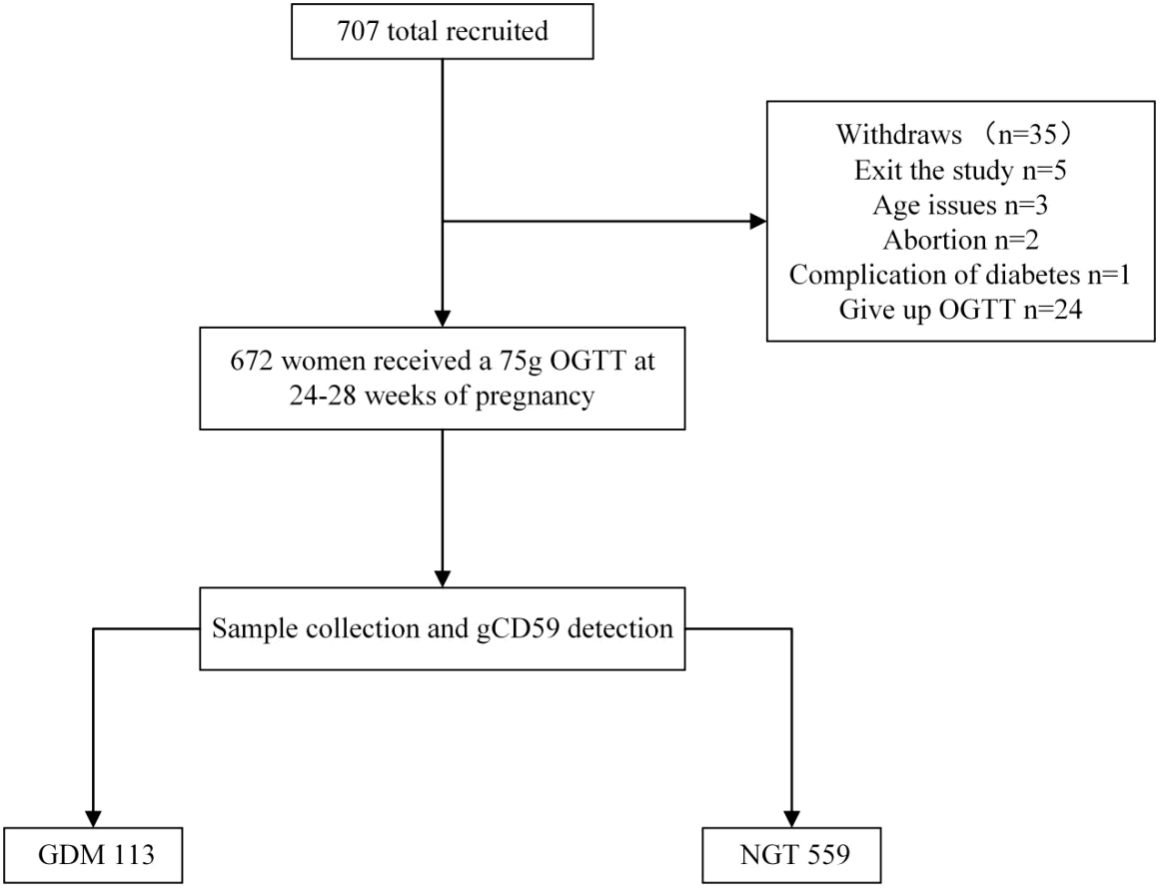
Flowchart. OGTT, Oral glucose tolerance test; GDM, gestational diabetes mellitus; NGT, normal glucose tolerance.

This research was a prospective cohort study, as depicted in the accompanying flowchart (**Figure 1**). Enrolled were pregnant women visiting the obstetrics outpatient clinic at The Affiliated Suqian Hospital of Xuzhou Medical University between January 2022 and July 2023. Eligible participants met the following criteria: aged 20-40 years, gestational period within 24-28 weeks, ultrasound-confirmed singleton intrauterine pregnancy, and provision of informed consent. Exclusion criteria encompassed a history of type 2 diabetes, severe organic or primary diseases such as liver or kidney dysfunction, heart failure, stroke, or malignant tumors, Exclusion of persons with cognitive, affective, motor and communication disorders according to the CCAS/Schmahmann scale (23).

### 2.2 Research methods

Upon their initial consultation, all participants were registered with electronic health records into the Maternal and Child follow-up System (ZhenDing System). This system captured key metrics, including age, sampling date, gravidity, parity, systolic blood pressure(SBP),diastolic blood pressure(DBP), as well as height and weight. Body Mass Index (BMI) was calculated in accordance with the classification standards of the World Health Organization (24). BMI classifications: a BMI less than 18.5 kg/m^2^ as underweight; a BMI ranging from 18.5 to 23.9 kg/m^2^ as normal weight; a BMI between 24 and 27.9 kg/m^2^ as overweight; and a BMI of 28 kg/m^2^ or higher as obesity. Participants were fasted for 8-14 hours to prepare for clinical laboratory assessments. At the first visit and during the 24-28 week, Peripheral venous blood was drawn from the forearm to measure an array of clinical parameters, including Hemoglobin (Hb), Ferritin, Alanine aminotransferase (ALT), Aspartate aminotransferase (AST), Blood urea nitrogen (BUN), Creatinine (Cr), Uric acid (UA), Triglycerides (TG), Total Cholesterol (TC), Thyroid Peroxidase Antibody (TPOAb), Free Thyroxine (FT4), and Thyroid Stimulating Hormone (TSH).Use of colour Doppler ultrasound scan to assess the subject’s gestational week according to the International Society of Ultrasound in Obstetrics and Gynaecology(ISUOG) guidelines (25).

For the OGTT administered during 24-28 weeks of gestation, a 75g glucose solution, prepared from anhydrous glucose powder provided by Shandong Qidu Pharmaceutical Co., Ltd., was consumed following a fasting of 8-14 hours. Then, 75 grams of anhydrous glucose powder was mixed into 300 milliliters of water and promptly consumed within a 5-minute timeframe. Blood samples were subsequently collected from the elbow vein at fasting, and then 1 and 2 hours post-glucose intake. Blood glucose levels were analyzed using the plasma glucose oxidase method. Adhering to IADPSG diagnostic criteria, GDM was diagnosed when any one or more of the following indexes were met: fasting blood glucose levels greater than 5.1 mmol/L, blood glucose levels exceeding 10.0 mmol/L one hour post-glucose consumption, or blood glucose levels surpassing 8.5 mmol/L two hours after glucose intake (26).

Sample Collection: Blood samples were collected from all subjects during the 24-28 week gestation period after fasting for 8-14 hours. Each sampling involved the collection of 5 ml of venous blood using Ethylenediaminetetraacetic acid (EDTA) tubes, followed by centrifugation at 3000/min for 10 minutes at room temperature to separate the plasma. For gCD59 quantification, plasma samples (1 ml each) were reserved for gCD59 analysis. Each of these gCD59 plasma samples was subdivided into two aliquots of 500 µL and securely stored in a -80° freezer. To maintain confidentiality, all laboratory specimens were anonymized and tested in the central laboratory of our hospital. Throughout the testing process, laboratory personnel remained blinded to participants’ glycemic status to ensure objectivity.

Assessment of plasma gCD59: plasma concentrations of gCD59 were measured by a highly sensitive and specific human enzyme-linked immunosorbent assay (ELISA) kit (TSZ, USA) and refer to the approach developed by Ghosh et al. (27). Sample concentrations were obtained by a fourparameter logistic curve-fit, with a minimum detectable gCD59 concentration of 0.025 SPU((1SPU = 1 ng/ml). Intra- and inter-assay coefficients of variability were 4.9% and 5.4%, respectively.

### 2.3 Statistical methods

Data analyses were performed utilizing SPSS version 26.0 and R software version 4.3.1. All variables were subjected to the Kolmogorov-Smirnov normality test, data conforming to normal distribution were expressed as mean) $\bar{\text{X}}$ ± standard deviation(S) with the two independent samples t-test applied for comparisons between groups. Continuous variables that deviated from a normal distribution were described using median(*M*) and interquartile ranges(*P*25, *P*75), with the Mann-Whitney *U* test applied for comparisons between groups. Categorical data were expressed as frequencies(n) and percentages(%), with the Chi-square (χ^2^) test employed for intergroup analysis. To delineate the risk factors for GDM, univariate and multivariable binary logistic regression analysis was conducted. The diagnostic performance of plasma gCD59 for GDM was quantitatively assessed through the area under the curve (AUC) and 95% confidence intervals (*CI)* determined in the receiver operating characteristic (ROC) curve analysis. All tests were two-sided and *P* < 0.05 was considered statistically significant.

## 3 Results

### 3.1 Comparative analysis of general and biochemical indicators

In this investigation, 707 subjects were initially engaged. Subsequently, adjustments to the participant pool were made: five individuals dropped out, three were disqualified in age, two encountered miscarriages, one presented with complications of Type 2 Diabetes, and 24 abstained from the OGTT. These modifications resulted in a final cohort of 672 subjects. Within this group, 113 were classified under the GDM category, establishing a GDM incidence rate of 16.8% (**Figure 1**). The initial comparative analysis of baseline characteristics revealed that the GDM group exhibited significantly higher values of weight, BMI, SBP, DBP, and TG, compared to the Non-Gestational Diabetes Mellitus (NGT) group (*P* < 0.05). Glycemic indicators including fasting glucose, and 1-hour and 2-hour post-glucose intake levels were notably elevated in the GDM group versus the NGT group (*P* < 0.001) (**Table 2**).

**Table 2:** Comparison of general and biochemical data between GDM and NGT groups [M (*P*25, *P*75)] or n(%).

Baseline characteristics	GDM(n=113)	NGT(n=559)	*P* Value
FPG(mmol/L)	5.21(4.93,5.46)	4.52(4.26,4.73)	<0.001
1h glucose(mmol/L)	9.20(7.21,10.31)	7.22(6.32,8.16)	<0.001
2h glucose(mmol/L)	8.07(6.63,8.99)	6.30(5.64,7.07)	<0.001
Age(years)	30(27,33)	30(27,33)	0.40
Gravidity	2(1,3)	2(1,3)	0.12
Parity	2(1,2)	1(1,2)	0.15
Height(cm)	162(160,165)	163(160,165)	0.53
Weight(kg)	62.5(57.25,70)	58(52.5,65)	<0.001
BMI(kg/m^2^)	24.06(22.04,24.07)	21.87(20,21.88)	<0.001
SBP(mmHg)	112(106,124)	110(104,118)	<0.001
DBP(mmHg)	73(68.25,80)	69(65,74)	<0.001
Ferritin(ng/ml)	15.7(8.7,26.3)	13.5(7.8,23.9)	0.53
Hb (g/L)	119(112,124)	118(112,124)	0.68
BUN(mmol/L)	2.78(2.5,3.46)	2.77(2.31,3.28)	0.19
Cr(mmol/L)	45(40.6,49)	45(40.5,49)	0.68
UA(umol/L)	245.7(209.63,297.58)	240.1(203.7,282.2)	0.33
TG(mmol/L)	1.79(1.34,2.9)	1.56(1,2.75)	0.01
TC(mmol/L)	4.65(4.03,6.4)	5.18(4.18,6.61)	0.13
ALB(g/L)	37.3(35.35,41.35)	38.3(35.7,41.8)	0.18
ALT (U/L)	12.35(8.68,17.03)	12(9,16.8)	0.76
AST (U/L)	15.65(13.68,18.48)	16.1(14,18.8)	0.45
TPOAB(IU/mL)(+)	9(9.96%)	26(4.65%)	0.15
FT4(pmol/L)	12.65(11.4,14.6)	12.6(11.4,13.8)	0.72
TSH(uIU/mL)	1.94(1.28,2.85)	1.83(1.25,2.54)	0.15
gCD59(SPU)	1.49(0.90,2.33)	0.87(0.39,1.62)	<0.001

GDM, gestational diabetes mellitus; NGT, normal glucose tolerance; FPG, fasting plasma glucose; BMI, body mass index; SBP, systolic blood pressure; DBP, diastolic blood pressure; Hb, hemoglobin; BUN, blood urea nitroge; Cr, Creatinine; UA, uric acid; TG, triglyeride; TC, total cholesterol; ALB, albumin; ALT, alanine aminotransferase; AST, aspartate aminotransferase; TPOAB: FT4, free theroxine; TSH, thyroid stimulating hormone.

### 3.2 Distribution of gCD59 in two groups

**Figure 2:**
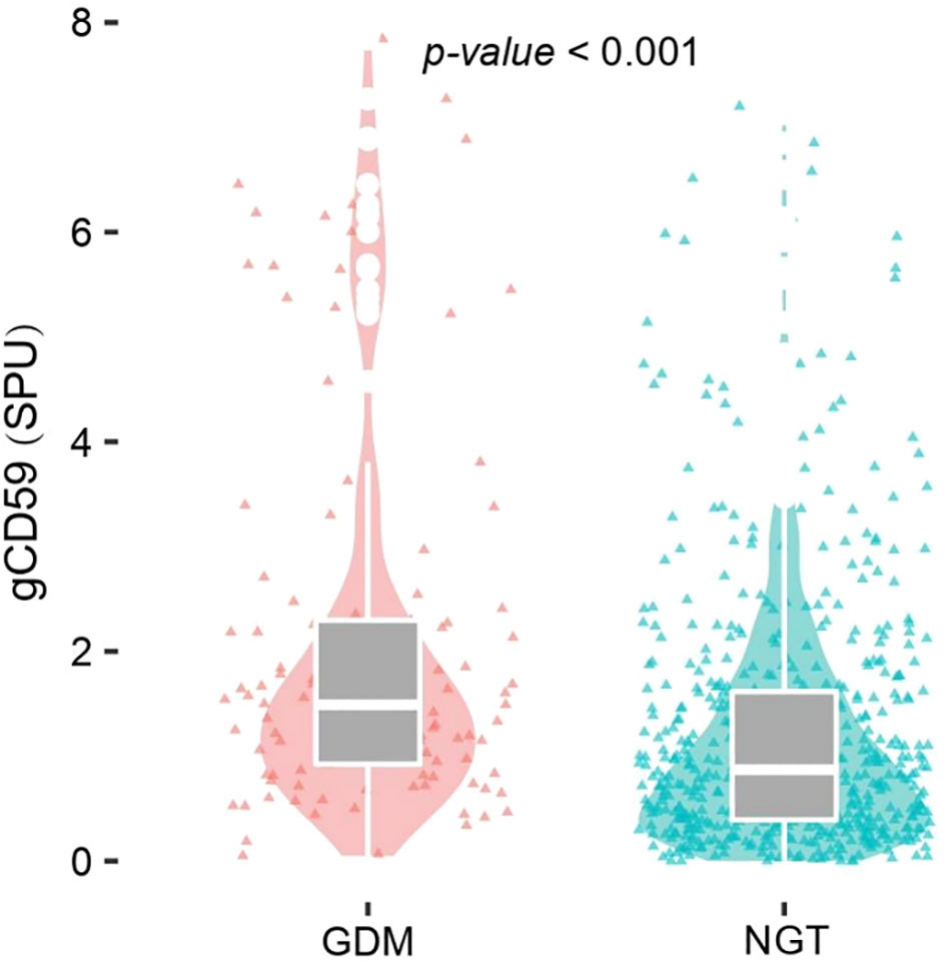
Ditribution of gCD59 in the GDM group and NGT group.

The analysis demonstrated a significant elevation of median gCD59 levels in the GDM group, compared with the NGT group (1.49 SPU vs 0.87SPU) (*P*<0.001)(**Figure 2**).

### 3.3 Identification of Risk Factors for GDM

With the occurrence of GDM as the dependent variable, gCD59, age, pregnancy, weight, BMI, SBP, DBP, TSH, Ferritin as the independent variables, the initial univariate regression analysis highlighted significances of gCD59, weight, BMI, DBP, and TSH (*P* < 0.05) (Model 1). Upon further adjustment for gravidity, weight, BMI, SBP, and DBP, the results of the multivariate regression analyses found that gCD59 (*OR*=1.417) and DBP (*OR*=1.050) persisted as significant independent risk factors for GDM (Model 2). Subsequent analysis with adjustments for age, TSH, and Ferritin multivariate identified gCD59 (*OR*=1.572), DBP (*OR*=1.047), and TSH (*OR*=1.314) as independent risk factors for GDM. Notably, every increment of 1SPU in gCD59 was associated with a 1.57-fold increase in GDM risk. These findings underscored the nuanced interplay of physiological factors with GDM (Model 3), offering insights for future preventative and diagnostic strategies (**Table 3**).

**Table 3:** Univariate and Multivariate Logistic Regression Analysis of Risk Factors for GDM.

variables	Model 1		Model 2		Model 3	
	OR (95%*CI*)	*P*	OR (95%*CI*)	*P*	OR (95%*CI*)	*P*
gCD59	1.418(1.254-1.604)	<0.001	1.417(1.244-1.614)	<0.001	1.572(1.347-1.834)	<0.001
Gravidity	1.031(0.898-1.183)	0.67	0.969(0.834-1.126)	0.679	0.918(0.765-1.102)	0.359
Weight	1.040(1.020-1.06)	<0.001	0.992(0.936-1.051)	0.786	1.012(0.952-1.075)	0.699
BMI	1.123(1.066-1.183)	<0.001	1.121(0.955-1.316)	0.163	1.079(0.913-1.275)	0.372
SBP	1.031(1.013-1.049)	0.001	0.989(0.964-1.014)	0.393	0.981(0.955-1.008)	0.174
DBP	1.055(1.031-1.079)	<0.001	1.050(1.017-1.085)	0.003	1.047(1.012-1.083)	0.009
Age	1.016(0.971-1.063)	0.491			1.027(0.967-1.092)	0.381
TSH	1.215(1.03-1.433)	0.021			1.314(1.095-1.577)	0.003
Ferritin	0.999(0.990-1.009)	0.894			0.995(0.984-1.007)	0.416

Model 1: Unadjusted factors; Model 2: Adjusted for gravidity, weight, BMI, systolic pressure, diastolic pressure; Model 3: Further adjusted for age, TSH, and Ferritin based on Model 2.

### 3.4 Diagnostic potential of gCD59 for GDM

**Figure 3:**
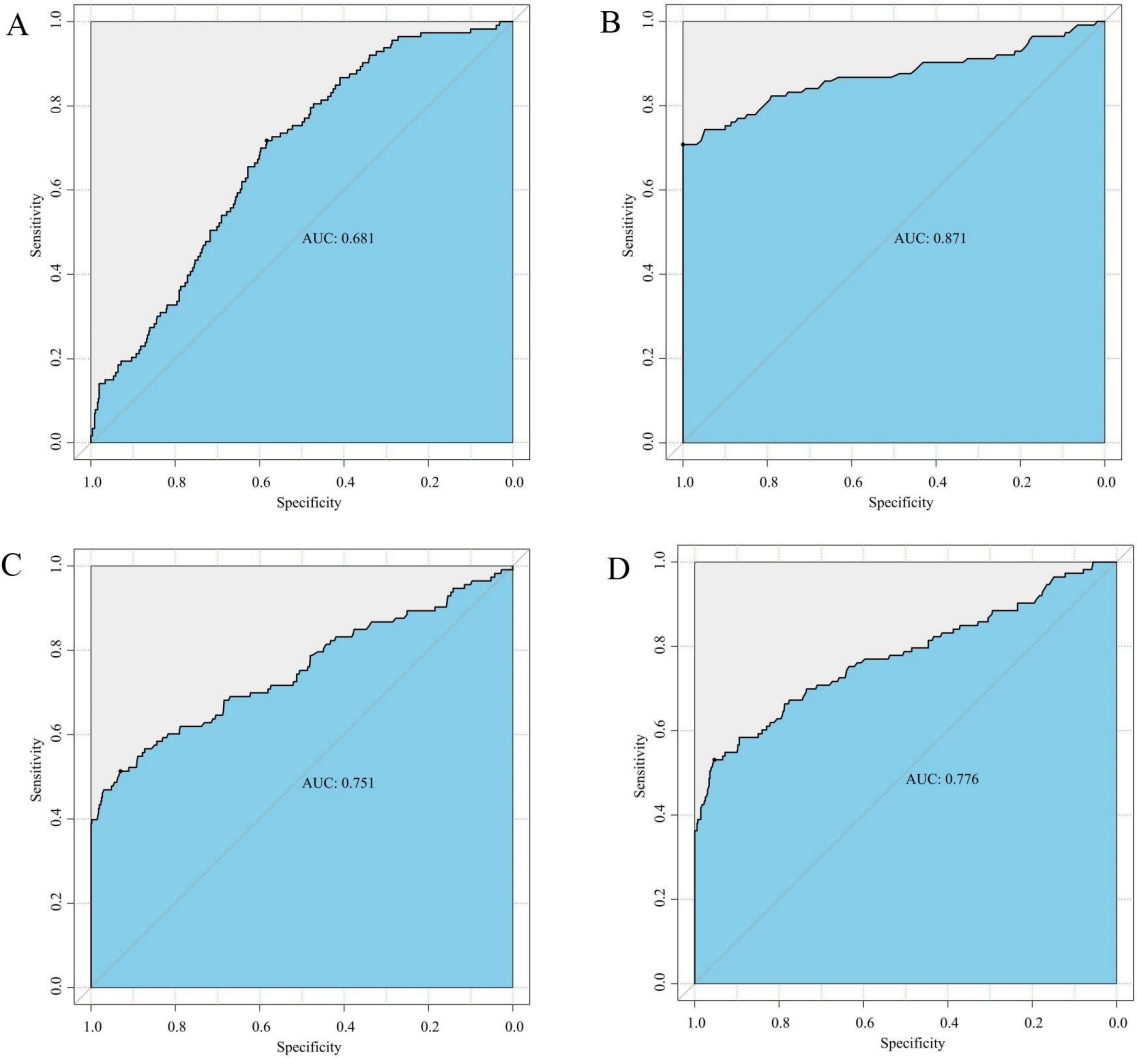
**(A)** ROC curve for gCD59 in diagnosis of GDM; **(B)** ROC curve of gCD59 in combination with fasting blood glucose (5.1 mmol/L) for the diagnosis of GDM; **(C)** ROC curve of gCD59 in combination with 1-hour blood glucose (10.0mml/L) for the diagnosis of GDM; **(D)** ROC curve of gCD59 in combination with 2-hour blood glucose (8.5mmol/L) for the diagnosis of GDM.

ROC curve analysis revealed an AUC of 0.681 (95%*CI*: 0.583-0.717), indicating a sensitivity of 71.7% and a specificity of 58.3% for gCD59 in predicting GDM occurrence (**Figure 3A**). We conducted a detailed analysis in combination with a single diagnostic threshold during the OGTT. Our results showed that gCD59, in combination with fasting blood glucose, effectively diagnosed the occurrence of GDM, the area under the ROC curve (AUC) was 0.871 (95% *CI*: 0.708-1.000) (**Figure 3B**). Similarly. when combination with 1-hour and 2-hour post-glucose levels as diagnostic thresholds, gCD59 maintained its strong diagnostic ability, with AUCs of 0.751 (95%*CI*: 0.513-0.930) and 0.776 (95%*CI*: 0.531-0.953), respectively (**Figures 3C, D**).

**Figure 4:**
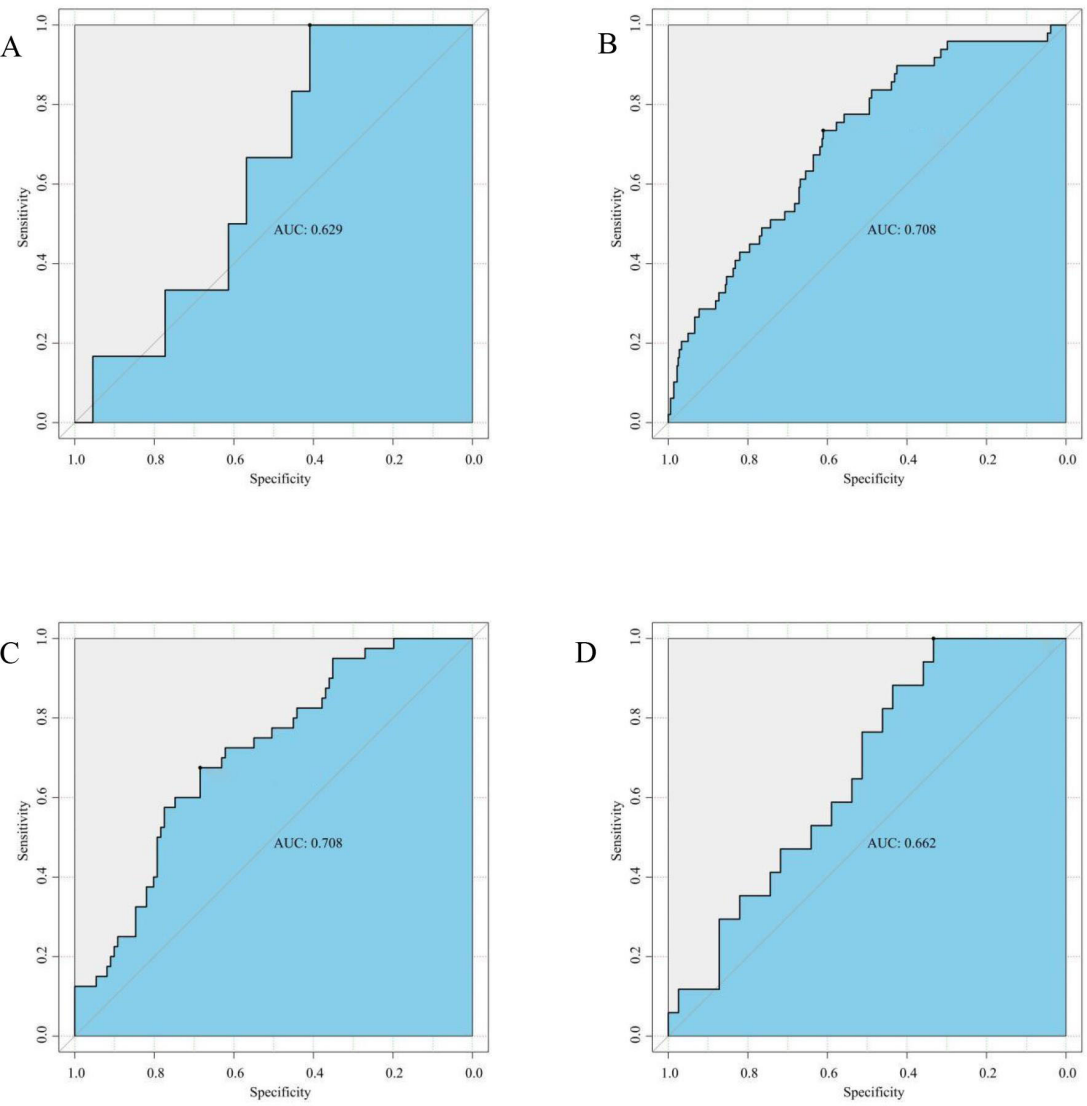
**(A)** ROC Curve for gCD59 in diagnosis of GDM among Underweight Participants (BMI < 18.5 kg/m2,n(GDM n=6,NGT n=44); **(B)** ROC Curve for gCD59 in diagnosis of GDM among Normal Weight Participants (18.5 kg/m^2^ ⩽ BMI ⩽ 23.9 kg/m2,GDM n=49,NGT n=365); **(C)** ROC Curve for gCD59 in diagnosis of GDM among Overweight Participants (24 kg/m^2^ ⩽ BMI 27.9 kg/m^2^,GDM n=41,NGT n=111); **(D)** ROC Curve for gCD59 in diagnosis of GDM among Obese Participants (BMI ≥28 kg/m2, GDM n=17,NGT n=39).

We further investigated diagnostic abilities of gCD59 across different BMI categories. In the underweight cohort, gCD59 diagnosed GDM with an AUC of 0.629 (95%*CI*: 0.409-1.000) (**Figures 4A**). This diagnostic capability was more pronounced in the normal and overweight groups, with AUCs of 0.708 (95%*CI*: 0.610-0.735 and 0.675-0.685, respectively) (**Figures 4B, C**). However, in the obese category, a slight reduction in diagnostic accuracy was noted, with an AUC of 0.662 (95%*CI*: 0.333-1.000) (**Figure 4D**).

## 4 Discussion

The quest for accessible and efficient biomarkers is ongoing, given the limitations of the OGTT in diagnosing GDM. Notably, gCD59 has emerged as a promising candidate in this regard. Our study underscores a significant elevation of gCD59 in the GDM cohort among the Chinese population, indicating its potential as an independent biomarker for GDM diagnosis as well as an independent risk factor for the development of GDM. The observed efficacy of gCD59, with an AUC of 0.681 and balanced sensitivity and specificity rates. In combination with fasting glucose, gCD59 effectively diagnosed GDM with higher AUC of 0.871, reinforces its utility in clinical settings.

Involving 672 participants and adhering to the IADPSG criteria, this study discerned a GDM prevalence of 16.8% (113 patients), reflecting the escalating trend of GDM in China. Contemporary epidemiological findings reveal that the incidence has already exceeded 15% (28). The study indicates that the incidence of GDM among the participants is reflective of the general condition of pregnant women across China. This research aligns with the increasing body of evidence pointing to maternal age, obesity, hypothyroidism, and elevated ferritin levels as risk factors in GDM pathogenesis (20, 29–31). Our findings particularly noteworthy that age did not significantly differ between groups, potentially due to the lower proportion of older mothers (approximately 9% of the total population.), hinting at the need for broader demographic studies. The multivariate regression analysis identified gCD59, DBP and TSH as independent predictors for GDM. Notably, gCD59 had the highest OR value. An increment of 1SPU in gCD59 was associated with a 1.57-fold increase in GDM risk. As a complement regulatory protein, CD59 inactivation leads to a loss of inhibition against the membrane attack complex (MAC), potentially escalating MAC deposition. This mechanism may exacerbate diabetes and its sequelae (18, 32). The study underscored a marked elevation in gCD59 within the GDM group, indicating its potential role in the etiology and progression of GDM among pregnant women. Further investigation is warranted to elucidate the underlying mechanisms.

Bogdanet et al. (21) have reported an AUC of 0.65 for gCD59 in GDM prediction, closely aligning with the 0.68 in our study. However, in this study, the median gCD59 level was notably lower compared to those reported by Bogdanet et al. (1.49 SPU versus 2.6 SPU). A critical analysis revealed that the population in the study of Bogdanet et al. was characterized by a higher median age of 34.8 and a median BMI of 28.7, markedly surpassing those in our cohort. These differences in age and BMI might have contributed to the variance in gCD59 level between the two groups. Ghosh et al. (22) utilized a two-step method for diagnosing GDM. Their findings demonstrated that gCD59 had a significant predictive accuracy for GDM, with an AUC of 0.92 (95% *CI*: 0.77-0.91). Remarkably, the diagnostic efficacy of gCD59 was enhanced when sampling time was performed at the same time point. This enhancement can be ascribed to several factors. Primarily, the two-step method plays a crucial role. Literature suggests that the prevalence of GDM identified using the one-step method is threefold compared to the two-step method (33). Additionally, the elevated diagnostic threshold in the two-step method tends to include participants presenting higher blood glucose levels. In this study, we expanded the racial diversity of participants, and included cases of multiple pregnancies as a high-risk group for GDM. By employing more stringent inclusion criteria, the diagnostic utility of gCD59 in GDM was significantly enhanced. Our results showed that gCD59 in combination with fasting glucose effectively diagnosed GDM with a higher AUC of 0.871 (95% CI: 0.708-1.000). Given its nature as a glycosylated protein, gCD59 more accurately mirrors initial glucose levels. Notably, prior research indicates that relying solely on elevated fasting blood glucose levels for GDM diagnosis could potentially obviate the need for OGTT in upwards of 50% of the population (34). This underscores the potential of elevated fasting glucose level as a preliminary screening marker for GDM. Nevertheless, how to determine an optimal fasting glucose threshold that balances sensitivity and specificity remains a subject of ongoing debate (35, 36).

Given the established association between BMI and GDM, our study stratified participants into underweight, normal, overweight, and obese based on BM to assess the predictive abilities of gCD59 in these subgroups. It revealed a general increasing trend of gCD59 diagnostic values with BMI, with AUC values of 0.629, 0.708, 0.708, and 0.662 respectively. However, an unexpected decrease in AUC was observed in the BMI > 28 kg/m²subgroup, which differs from the findings of Bogdanet et al. that AUC values increased progressively with BMI. they found that the predictive value of gCD59 for GDM increased progressively with increasing BMI, and the AUC reached the highest (AUC=0.96) in the BMI 40 kg/m^2^ group, this difference may be due to the different BMI distribution of the enrolled population. The median BMI of the enrolled population in this study was 24.06, significantly lower than that of Bogdanet et al. Given the limited sample size of this subgroup which included only seven pregnant women with a BMI above 35 kg/m^2^, comprehensive analysis of higher BMI subgroups was not feasible. Therefore, future studies, incorporating a more extensive sample size, are imperative to elucidate the diagnostic accuracy of gCD59 across varied BMI categories.

The small sample size of the enrolment in this study and the lack of matching of the enrolled population may have biased the results. In addition, there was no specific information on maternal weight gain during pregnancy, which may have biased the results of the BMI subgroup analyses. The third is that a validation study in another prospective cohort with a different participant population is needed to confirm the sensitivity and specificity of the results of this study.

This study supports the potential of gCD59 as a diagnostic biomarker for GDM in a Chinese population. and further confirmation of the value of gCD59 in diagnosing GDM at different stages of pregnancy based on multicentre and larger sample size is needed in the future. The combination of other biological markers may also be considered for further analysis.

## Data Availability

The original contributions presented in the study are included in the article/supplementary material. Further inquiries can be directed to the corresponding authors.
